# The Interactive Functions of Questions in Embodied Collaborative Work

**DOI:** 10.3389/fpsyg.2021.704275

**Published:** 2021-08-30

**Authors:** Lucas M. Bietti, Federico U. Bietti

**Affiliations:** ^1^Department of Psychology, Norwegian University of Science and Technology, Trondheim, Norway; ^2^CITERES-CoST, University of Tours, Tours, France

**Keywords:** questions, functions, embodied collaboration, work, interaction

## Abstract

Researchers have been interested in the investigation of the interactive functions of questions in conversational contexts. However, limited research has been conducted on the interactive functions of questions in embodied collaborative work, that is, work that involves the manipulation of physical objects. This study aimed to identify the interactive functions of questions in embodied collaborative work. To do so, we conducted a systematic qualitative analysis of a dataset of 1,751 question-answer sequences collected from an experimental study where pairs of participants (*N* = 67) completed a collaborative food preparation task. The qualitative analysis enabled us to identify three functions of questions: *anticipation questions*, *exploration questions*, and *confirmation questions*. We have discussed in this study how the types of questions identified are associated with: (i) the accomplishment of interactional goals and (ii) complementary temporalities in the collaborative activities.

## Introduction

Questions are one of the most important linguistic and embodied resources giving structure to social interactions, from human interaction in every day (Fox and Thompson, 2010) and institutional (Murtagh et al., 2013) settings to human-animal interaction (Mondémé, 2020) and human interactions with virtual agents (Liao et al., 2018) and social robots (Pitsch et al., 2017). Questions have specific interactive functions, including requests for information (Couper-Kuhlen, 2012) and confirmation (Hayano, 2014); seek for help (Erkelens et al., 2021) and agreement (Heritage and Raymond, 2005); perform assessments (Lindström and Mondada, 2009); make suggestions, proposals, and offers to others (Stevanovic, 2012); and make other-initiated conversational repairs (Schegloff, 2000).

Stivers and Enfield (2010) proposed a basic differentiation between content (Q-word) questions (e.g., What did you eat for breakfast?), polar questions (e.g., Did you go to the movies yesterday?), and alternative questions (e.g., Are we going for a walk or do you prefer to stay at home?). Such initial differentiation responds to lexical, morphological, syntactical, and prosodic features (Couper-Kuhlen, 2012) that vary depending on the particularities of language families investigated, allowing their (almost) effortless identification in face-to-face conversations.

Questions embedded in embodied collaborative work (e.g., assembling IKEA furniture with a partner) rely on the coordination of bodily resources such as manual gestures, gaze, posture, and facial expressions to achieve shared goals. The joint accomplishment of shared goals goes beyond the coordination of verbal and non-verbal behaviors in a synchronized manner over time. Collaboration influences the action and planning of interacting partners and shapes interactive outcomes, such as when partner A asks “What should we do with this (while pointing to a bag of flour)?” B replies: “we must put half of that in this bowl (while touching the bowl),” and A acknowledges B: “Alright, I see it now.” This short question-answer sequence illustrates how the intentions, plans, and goals of each interacting partner come into play in embodied collaborative work.

This study aimed to identify the interactive functions of questions in embodied collaborative work involving the manipulation of physical objects. To do so, we conducted a qualitative analysis of a dataset of 1,751 question-answer sequences collected from an experimental study where pairs of participants (*N* = 67,134 participants) completed a collaborative food preparation task. Case studies have been used to investigate the interactive functions of questions in embodied collaborative work (e.g., Bietti and Baker, 2018a,b). To our knowledge, no research has provided a systematic identification of the interactive functions in embodied collaborative work where the manipulation of physical objects becomes essential in a large sample.

First, we reviewed the literature focused on the investigation of the interactive functions of questions in conversational contexts. Second, we described the experimental task where the 1,751 question-response sequences analyzed here were collected. Third, we presented a description of the methodological aspect of the study. Fourth, we identified the main types of questions observed in the question-response sequences and showed their distribution in the dataset. Fifth, we presented illustrative examples of each of the type questions identified, including multimodal analyses of each question-response sequence. We finally discussed how the types of questions identified here are linked to the accomplishment of interactional goals and to complementary temporalities in the embodied collaborative activities.

## Background

Questions are recipient-designed and oriented toward interacting partners, that is, the speaker who poses the question assumes that the recipient possesses the information requested. The study of adjacency pairs (e.g., Sacks and Schegloff, 1973; Sacks et al., 1974; Schegloff, 2007) has enabled the systematic investigation of the interactive functions of question-response sequences in conversations. Adjacency pairs are units of conversation composed of two turns or pair parts (in the present case: Question ⇒ Answer); each pair parts have to come from a different speaker (A and B), be placed adjacently (1. A: Question ⇒ 2. B: Answer) unless separated by an insertion sequence (1. A: Question ⇒ 2. B. Request for clarification ⇒ 3. A: Answer [turns 2B and 3A is the insertion sequence] ⇒ 4. B: Answer) and the second pair part is identified as functionally related and relevant (Hutchby and Wooffitt, 2008) to the first pair part (e.g., A: “Where did we buy these glasses?” B: “At the flea market”). Questions (first pair part) impose special constraints on answers (second pair part). In engaging in such sequences, interacting partners impose constraints on one another and hold each other accountable, to produce coherent and intelligible courses of action in relation to relevant actions and events. Questions requesting for information (first pair part) may have multiple responses (second pair part). However, there are generally two types of responses to such questions, either accepting or declining the request (Schegloff, 2007). Accepting or declining the request for information involves different kinds of interactional work. Accepting the request comes with no cost for reputation and may even increase the reputation of the interacting partner providing the answer. In contrast, declining the request for information may lead to reputational risks that are often introduced by delays in responses (e.g., “well”) and followed by accounts aimed at mitigating the refusal (e.g., “well, I don’t remember exactly because I wasn’t paying attention when she gave the explanation”). Thus, accepting the request for information is considered to be a case of preferred response, whereas declining it is considered to be a case of dispreferred response (Heritage, 1984).

Researchers have investigated the interactive functions of questions in everyday conversations (Tannen et al., 2007; Bietti, 2010, 2013; Bietti and Galiana Castello, 2013; Fox and Thompson, 2010; Rossano, 2010; Bova and Arcidiacono, 2013; Goodwin and Cekaite, 2013), children peer-to-peer conversations (Baucal et al., 2013; Stivers et al., 2018), clinical populations (Goodwin, 1995; Antaki, 2013; Laakso, 2015; Anglade et al., 2021), medical consultations (Heath, 1986; Heritage and Robinson, 2006; Murtagh et al., 2013; Mayor and Bietti, 2017), police interrogations (Stokoe and Edwards, 2008; van Charldorp, 2014), job interviews (Bangerter et al., 2014; Brosy et al., 2016), classroom interactions (Margutti and Drew, 2014; Hosoda, 2016; Ishino, 2017), service encounters (Fox, 2015; Mondada and Sorjonen, 2016; Lindström et al., 2019), helpline services (Butler et al., 2010), guided tours (Mondada, 2017), team meetings at the workplace (Svennevig, 2012), and political (Gialabouki and Pavlidou, 2019) and immigration interviews (Channon et al., 2018). Several of these studies adopted a multimodal perspective to the analysis of question-response sequences (e.g., Stivers and Rossano, 2010).

The study of the interactive functions has also been studied in embodied collaborative work involving the manipulation of physical and digital objects in a variety of contexts, including operating theaters (Hindmarsh and Pilnick, 2007; Bezemer et al., 2016), design (Bietti et al., 2016; Bietti and Baker, 2018a), and architectural (Murphy, 2004; Bietti and Baker, 2018b) studios, and radio stations (Risberg and Lymer, 2020). These studies have shown the multiple ways in which questions redirect the attention of team members to objects and actions. For example, Bietti and Baker showed that reminders in the form of questions (e.g., A: “When did they tell us the deadline for this was? Because I am not sure whether it was Friday or Saturday,” B: “It was Friday,” A: “Great, thanks!”) redirected the attention of professional designers to objects (e.g., computer screens) and actions (e.g., product delivery to client) in the design studio. Bietti and Baker found that questions acting as reminders scaffolded future planning and collaborative decision-making among professionals designers at the design studio. Research on the interactive functions of questions at the workplace has been conducted exclusively in real-world situations using case studies. To our knowledge, no research has provided a systematic identification of the interactive functions of questions in embodied collaborative work using a large sample of participants. This is important if we are interested in gaining a better understanding of the multiple ways in which different types of questions structure social interaction in collaborative work beyond specific cases, activities, and settings.

## The Current Study

This study aimed to identify the interactive functions of questions in embodied collaborative work involving the manipulation of physical objects. To do so, we examined 1,751 question-response sequences in a dataset taken from an experimental study on the cultural transmission of cooking skills. In part of the experiment from where we extracted the question-response sequences, participants were asked to prepare the highest number possible of ravioli in pairs (refer to description of Task below). The data collected were in French. The only constraint involved in the collaborative ravioli-making task was time duration (refer description of Task below). Hence, group members could freely interact while making the ravioli, as it would occur in a comparable real-world situation.

## Methods

### Participants

A total of 134 participants (67 pairs; 76 men) were recruited from the student population of the University of Neuchâtel, Switzerland (age M = 23.2; SD = 4.04). They were fluent speakers of French and reported having limited previous cooking experience. They had previous practice of simple skills like combining and heating ingredients but had not mastered more complex skills (e.g., preparing a pie from scratch). Participants received 25 CHF compensation each for half an hour of their time along with an incentive of 0.25 CHF per pair for each produced ravioli of good quality.

### Task

The task consisted of two kinds of sessions, namely, performance sessions and transmission sessions. The question-response sequences analyzed in this study were collected from performance sessions only where participants prepared ravioli together in pairs. Their goal was to produce as many good-quality ravioli as possible in 10 min. Each pair had at their disposal a ball of 150 g of dough, 200 g of filling made of ricotta cheese and concentrated tomato paste (for easy detection of leaks and imperfections when evaluating ravioli quality), a 24-hole ravioli mold with zigzag sealing for easy release, a pasta maker, a rolling pin, a cutting board, 2 pizza cutters, 2 knives, 4 teaspoons, 2 kitchen cloths and kitchen paper, 250 g of flour, and a stopwatch. The collaborative food preparation task had 10 consecutive phases listed as follows: (1) divide the dough in two, (2) add flour to the dough, (3) use rolling pin to flatten the dough, (4) use pasta maker to flatten the dough, (5) cut dough in half, (6) put the first layer of dough over the mold, (7) add the filling, (8) cover with second layer of dough, (9) flip over the mold, and (10) cut ravioli. Immediately after the time was up, the ravioli was assessed by the experimenter.

### Coding

The identification of questions followed the coding scheme for question-response sequences in conversation developed by Stivers and Enfield (2010). Their coding scheme is empirically grounded in a comparative project on question-response sequences in ordinary conversation in 10 languages. The coding scheme considers the interactive functions of questions as well; from information and confirmation requests, assessments, and suggestions to other initiation of repairs (e.g., What?) and rhetorical questions. It also accounts for the question-response sequences in which the answer corresponds to a visible action (e.g., head shakes, nods, shrugs, pointing gestures, and eyebrow flash) which is regarded as a relevant response. This is an essential feature for the throughout coding of question-response sequences in embodied collaborative work.

In French, content (Q-word) questions include interrogative pronouns such as qui/who, que/what, lequel/which, où/where, quand/when, and combien/how much. They can be followed by noun phrases, which form a syntactic constituent. Polar questions can be accompanied by a finite verb, a negative adverb, adverb of frequency, a clitic, or a subject. In alternative questions, each of the alternatives in the question is stressed. Any positive polar question can be turned into an alternative question by adding ou/or and the interrogative final particle hein/not. Declarative syntax ending with an interrogative intonation is the more frequent way in which questions are composed in French (Mondada, 2017).

### Categorization

We started the categorization procedure following well-established interactive functions of questions (e.g., request for information, request for confirmation, and seek for help) reported in the literature. Data were transcribed in InqScribe^TM^ for later synchronization with multimodal annotations in ChronoViz (Fouse et al., 2011). Transcriptions followed standard conventions in conversation analysis (Jefferson, 2004; Mondada, 2018). The synchronization of transcripts with multimodal annotations facilitated the familiarization with the data.

## Results

The familiarization with the data and the initial coding resulted in the differentiation between questions related to the collaborative task and questions that were not. We found that 84% (*n* = 1,521) of the 1,751 question-response sequences initially identified were task-related. New rounds of coding and revision of codes led to the categorization of the questions opening the 1,521 question-response sequences into three distinguishable themes. The interactive functions of questions in the collaborative food preparation task were as follows: (1) anticipation questions, (2) exploration questions, and (3) confirmation questions. *Anticipation questions* were requests for information about embodied actions that participants should perform in the next phases of the collaborative task while completing the present phase. *Exploration questions* were requests for information about embodied actions that the participant posing the question should execute to successfully progress in the ongoing phase of the task. *Confirmation question*s were requests for confirmation about embodied actions that were just performed or about to be performed in the ongoing phase of the task. Anticipation questions corresponded for 26% (*n* = 394), exploration questions corresponded for 41% (*n* = 618), and confirmation questions corresponded for 32% (*n* = 491) of all the task-related questions that initiated the 1,521 question-response sequences that we analyzed. Twenty percent of the 1,521 question-response sequences (*n* = 304) were double-coded. Inter-rater agreement for the three types of questions was very good (kappas ≥ 0.79). Disagreements across coders were resolved through discussion.

Below we present an example and description of each type of question-response sequence taken from the dataset to illustrate how they operated in practice.

### Anticipation Questions

Example 1 shows the coordination of an anticipation question with embodied actions and transition phases in the collaborative task (refer Methods for description of task phases).

**FIGURE 1:**
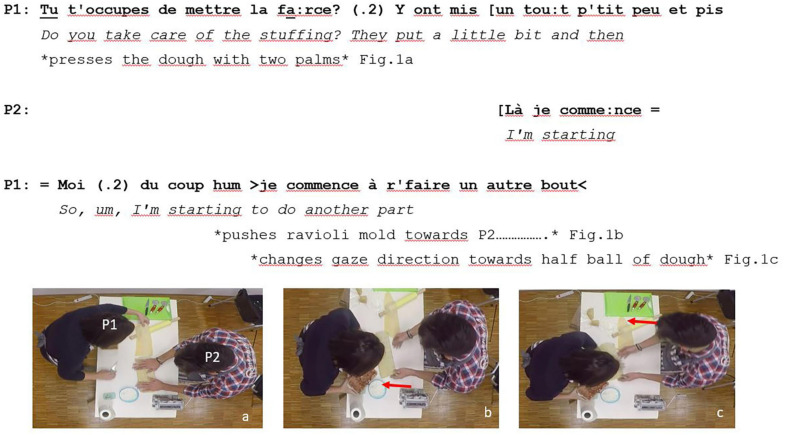
Example of anticipation question and coordination of embodied actions.

P1 made a polar question (1) acting as an anticipation question. This is the first element of the adjacency pair. He made it while pressing the first layer of the already flatten dough against the ravioli mold with his hand palms (Figure 1A). The response of P2 (2), accepting the request of P1 was the second element of the adjacency pair. Almost immediately P1 told P2 what he planned to do afterward (3), in the next phase of the collaborative task. He did it while placing the ravioli mold closer to P2 (Figure 1B). The goal of the embodied action of P1 was to place the ravioli mold in the visual attention field of P1. Placing for action (Clark, 2003) and change in gaze directions of P1 toward the half ball of dough (Figure 1C) closed the question-response sequence initiated by the anticipation question (1). The change in gaze direction of P1 predicted what he planned to do next in order to flatten the second layer of dough to cover the first layer, to which P2 was about to add the filling.

### Exploration Questions

**FIGURE 2:**
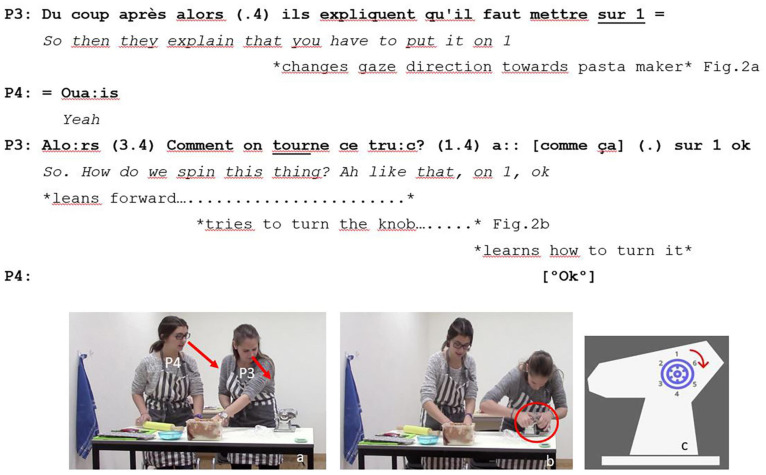
Example of exploration questions while learning how to use tools.

Example 2 presents an occurrence of an exploration question that one participant posed while performing an embodied exploration of the pasta maker. Dyads were instructed to carefully pass the dough through the pasta maker, which had two rollers that could be adjusted using the knob on the side. This allowed making the dough gradually thinner. Each time before passing the dough through the pasta maker, dyads had to turn the knob to switch levels (six levels, as shown in Figure 2C).

P3 changed the gaze direction toward the pasta maker while making explicit what they should do in the next phase of the task (1). The change in gaze direction of P3 toward the pasta maker directed the attention of P4 toward the same object (Figure 2A). Directing to the action of P3 (Clark, 2003) created a shared focus of visual attention and grounded mutual knowledge about the next phase of the task (Clark and Schaefer, 1989; Clark and Brennan, 1991). P4 agreed with the directing to action of P3 (2) and then P3 leaned forward to adjust the rollers of the pasta maker using the knob on the side (Figure 2C). She spent more than 5 s trying to figure out how the knob worked (3). The problem was that the knob had to be slightly pulled out; otherwise, it will not turn. P3 posed the content (Q-word) question because of having failed to learn how the knob worked (Figure 2B). The eureka moment came after a short silence: trial and error enabled her to learn how to turn the knob. P4 acknowledged P3 for solving the problem (4). Such acknowledgment overlapped with the embodied demonstration and response of P3 to her own exploration question (3).

### Confirmation Questions

Example 3 illustrates a question-response sequence about joint decision-making that was initiated by a confirmation question. The sequence shows participants deciding where they should trim the excess dough so it could successfully cover the entire surface of the ravioli mold.

**FIGURE 3:**
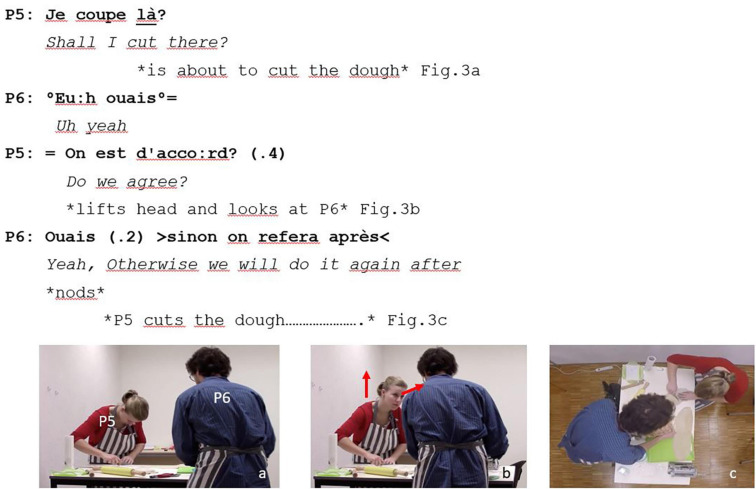
Example of confirmation question in joint decision-making.

The polar question of P5 (1) acted as a request for confirmation that opened the question-response sequence. P6 confirmed the decision of P5 to trim the excess dough where she had previously planned. However, he did it quietly using reduced speech volume (2) which did not seem to be very convincing for P5. As a result, P5 made a second confirmation question (3) as she had to be completely sure about how much excess dough she had to trim off. Having removed too much dough may have resulted in a shorter layer of dough that did not cover the complete surface of the mold. After a short silence, she lifted her head a looked at P6 (Figure 3B). The change in head position and gaze direction of P5 reinforced the accountability of P6 as he responded to her confirmation question (Goodwin, 1994; Bavelas et al., 2002; Stivers and Rossano, 2010). P5 trimmed off the excess dough immediately after receiving the affirmative response of P6 (4). She was already removing the excess dough when P6 added that they could do it again later (Figure 3C).

## Discussion

We investigated the interactive functions of content (Q-word) questions, polar questions, and alternative questions in a dataset of 1,751 question-response sequences collected in an experimental study in which 67 pairs of participants completed an embodied collaborative task in the laboratory. We did it using a collaborative food preparation task. The kind of task chosen aimed at increasing the ecological validity of the experimental design (Bietti et al., 2017, 2019). Food preparation is a social activity taught and learned across cultures and societies that currently attracts a lot of media attention. This is reflected in the increasing number of cookbooks that are sold annually, TV shows, online courses, and tutorials available on the subject. Food preparation is a meaningful real-world task that can boost creativity (McCabe and de Waal Malefyt, 2015) and have a positive impact on the self-esteem of people (Farmer et al., 2018). When food preparation occurs collaboratively, it strengthens social bonds by reinforcing family relationships, initiating and underpinning friendship (Wrangham et al., 1999). No previous observational or experimental study has examined the interactive functions of questions in embodied collaborative work in which the manipulation of physical objects played a central role in a large sample of participants.

We made a distinction between task-related and non-task-related questions (e.g., What do you study?) and observed that a significant proportion of questions were task-related. Then, we identified the interactive functions of task-related questions using the coding scheme developed by Stivers and Enfield (2010) and discovered that they had three main functions, that is, anticipation, exploration, and confirmation.

The three illustrative examples we presented showed the multiple ways in which the three types of questions were linked to the accomplishment of interactional goals: (i) planification of future collaborative activities (example 1), (ii) learning how an essential tool for the successful completion of the collaborative task works (example 2), and (iii) decision-making about an embodied action that would negatively affect group performance if it were done incorrectly. Interestingly, such goals referred to complementary temporalities in the activity. While example 1 (Figure 1) referred to a future phase of the task (e.g., add filling to the dough), examples 2 and 3 were related to the ongoing phase. Example 2 (Figure 2) was linked to an action (e.g., turn the knob) that dyads must perform to progress in the task (e.g., to flatten the dough in the pasta maker). Example 3 (Figure 3) was associated with an action, that if done inaccurately, would increase the risk of having a poor task performance (i.e., reduce the number of good quality ravioli produced). Questions presented in examples like 2 and 3 were coded differently because they reflected distinct levels of participants’ certainty. In cases like example 2, participants used trial-and-error methods to solve task-related problems. Such methods were not successful at first; therefore, participants decided to seek for help from their partner by making exploration questions. On the contrary, in cases like example 3, participants requesting confirmation from partners knew what the preferred course of action was. They suddenly decided to interrupt what they were doing to invite their partners to participate in the decision-making process. Thus, exploration questions presented a lower degree of participants’ certainty than confirmation questions. Anticipation questions were used to plan future phases of the collaborative task while still working on the current phase. On the contrary, exploration and confirmation questions often led to an interruption of the collaborative activities taking place within the task phase. Our results complement findings reported in ethnographic observations obtained through the careful analysis of video-recorded data in naturally occurring interactions.

The exploratory nature of the study did not allow us to define *a priori* hypotheses to test. For example, to have tested whether anticipation questions predicted higher performance in the number of good quality ravioli produced in comparison with exploration and confirmation questions would not have been appropriate considering that we did not have a condition for each type of question to calculate how each affected the performance individually. Another limitation of this study was the fact that we did not analyze the temporal distribution of questions in relation to their interactive functions throughout the embodied collaborative task. For example, it may have been the case that exploration questions were more frequent at the beginning of the task when participants had to figure out the functions of the cooking utensils that they had available. These are the kind of hypotheses we plan to test in further studies.

Future experimental studies will enable researchers to make predictions about what types of questions may lead to an increase in the quality of embodied collaborative work (e.g., performance) across tasks and large samples. This is important if we want to propose interventions to improve collaborative work involving the manipulation of physical and digital objects.

## Data Availability Statement

The data that support the findings of this study are openly available at the Open Science Framework (OSF): https://osf.io/5wdgt/.

## Ethics Statement

The studies involving human participants were reviewed and approved by Ethics Committee, University of Neuchâtel, Neuchâtel, Switzerland. The participants provided their written informed consent to participate in this study. Written informed consent was obtained from the individual(s) for the publication of any potentially identifiable images or data included in this article.

## Author Contributions

LB: conceptualization, data curation, formal analysis, investigation, methodology, project administration, and writing original draft. FB: investigation, formal analysis, and writing original draft. Both authors contributed to the article and approved the submitted version.

## Conflict of Interest

The authors declare that the research was conducted in the absence of any commercial or financial relationships that could be construed as a potential conflict of interest.

## Publisher’s Note

All claims expressed in this article are solely those of the authors and do not necessarily represent those of their affiliated organizations, or those of the publisher, the editors and the reviewers. Any product that may be evaluated in this article, or claim that may be made by its manufacturer, is not guaranteed or endorsed by the publisher.
